# Acetylation of histone H3K27 signals the transcriptional elongation for estrogen receptor alpha

**DOI:** 10.1038/s42003-020-0898-0

**Published:** 2020-04-07

**Authors:** Yujing Gao, Lijia Chen, Yali Han, Fangrui Wu, Wen-Si Yang, Zheng Zhang, Tong Huo, Yingmin Zhu, Chengtai Yu, Hong Kim, Mark Lee, Zhen Tang, Kevin Phillips, Bin He, Sung Yun Jung, Yongcheng Song, Bokai Zhu, Rui-Ming Xu, Qin Feng

**Affiliations:** 1 Center for Nuclear Receptors and Cell Signaling, Department of Biology and Biochemistry, University of Houston, Houston, TX 77204 USA; 2 Key Laboratory of Fertility Preservation and Maintenance of Ministry of Education, Department of Biochemistry and Molecular Biology, School of Basic Medical Sciences, Ningxia Medical University, 750004 Yinchuan, China; 3 National Laboratory of Biomacromolecules, CAS Center for Excellence in Biomacromolecules, Institute of Biophysics, Chinese Academy of Sciences, 100101 Beijing, China; 4 University of Chinese Academy of Sciences, 100049 Beijing, China; 5 Department of Pharmacology and Chemical Biology, Baylor College of Medicine, Houston, TX 77030 USA; 6 Department of Molecular and Cellular Biology, Baylor College of Medicine, Houston, TX 77030 USA; 7 Immunology & Transplant Science Center, Department of Surgery and Urology, Houston Methodist Research Institute, Houston, TX 77030 USA; 8 Department of Medicine, Weill Cornell Medicine of Cornell University, New York, NY 10065 USA; 9 Department of Biochemistry, Baylor College of Medicine, Houston, TX 77030 USA; 10 Department of Medicine, University of Pittsburgh, Pittsburgh, PA 15261 USA

**Keywords:** Histone post-translational modifications, Breast cancer

## Abstract

As approximately 70% of human breast tumors are estrogen receptor α (ERα)-positive, estrogen and ERα play essential roles in breast cancer development. By interrupting the ERα signaling pathway, endocrine therapy has been proven to be an effective therapeutic strategy. In this study, we identified a mechanism by which Transcription Start Site (TSS)-associated histone H3K27 acetylation signals the Super Elongation Complex (SEC) to regulate transcriptional elongation of the *ESR1 (ERα)* gene. SEC interacts with H3K27ac on *ESR1* TSS through its scaffold protein AFF4. Depletion of AFF4 by siRNA or CRISPR/Cas9 dramatically reduces expression of *ESR1* and its target genes, consequently inhibiting breast cancer cell growth. More importantly, a AFF4 mutant which lacks H3K27ac interaction failed to rescue *ESR1* gene expression, suggesting H3K27 acetylation at TSS region is a key mark bridging the transition from transcriptional initiation to elongation, and perturbing SEC function can be an alternative strategy for targeting ERα signaling pathway at chromatin level.

## Abstract (web summary)

Gao et al. study the relationship between AFF4 and acetylated histone H3K27 in the context of estrogen receptor alpha signaling. They use a series of pull down assays, genetic backgrounds, and global sequencing analysis and show that AFF4 binds to H2K27ac at promoters and influences the ER alpha signaling and breast cancer cell growth.

## Introduction

Breast cancer is the most commonly diagnosed cancer and the second leading cause of cancer death among women in the US. Approximately 70% of human breast tumors are estrogen receptor (ERα)-positive, making endocrine therapy which targets estrogen signaling pathway an effective therapy in treating ER-positive breast cancer^1^. However, since endocrine therapy resistance eventually occurs in ~50% of the patients, new therapies are urgently needed to overcome the resistance^2–4^. In many endocrine-resistant tumors, ERα remains to be essential for the tumor growth^5^. One mechanism of resistance is the development of ERα mutations. ERα mutations are very rare in primary and untreated breast cancer, but are detected in 20–50% of endocrine-resistant metastatic ER-positive breast tumors^6,7^. These mutations confer ERα protein’s ligand-independent transcriptional activity and therefore enable tumors to grow in a hormone-independent manner^8,9^. Currently there is no effective treatment for endocrine-resistant breast cancers with mutated ERα.

Eukaryotic gene expression is regulated by DNA-binding transcription factors and chromatin-associated epigenetic factors. GATA3 is one such transcription activator that induces *ESR1* gene expression^10^. However, GATA3 is currently undruggable because it lacks catalytic active sites for drugs to bind. We decided to identify chromatin-associated molecules that control the *ESR1* gene transcription. Targeting these regulatory molecules may shut down mutant *ESR1* gene expression and inhibit the growth of endocrine-resistant breast cancer.

Numerous studies have shown that the chromatin-associated epigenetic factors cooperate with post-translationally modified histone tails in regulating gene transcription. Among different histone modifications, histone acetylation is almost always correlated with transcriptional activation, regardless acetylation sites^11,12^. Histone acetylation can occur on all core histone proteins, including several highly conserved sites on histone H3 (K9, K14, K18, K23, K27), histone H4 (K5, K8, K12, and K16), and less conserved sites on histone H2A and H2B^13^. Acetylation of histone H3 on lysine 27 (H3K27ac) is the most studied histone acetylation, and is considered as the mark for active enhancers in many published ChIP-seq assays^14,15^. Interestingly, H3K27 acetylation does not solely occur on enhancers; a comparable level of H3K27ac can also be found next to proximal promoter/transcription start site (TSS) regions, where its function remains elusive. Very limited information is available on how TSS-specific H3K27 acetylation contributes to transcriptional activation.

In this study we identified AFF4, a scaffold protein of the Super Elongation Complex (SEC), as an important transcriptional elongation factor required for *ESR1* gene expression. Importantly, AFF4 functions as a key molecule in associating elongation machinery with K27-acetylated histone H3 on TSS. Depletion of AFF4 by CRISPR-Cas9 reduced the recruitment of transcriptional elongation factors and RNA polymerase II to the *ESR1* transcription start sites, decreased *ESR1* gene expression, and inhibited the growth of ER-positive breast cancer cells.

## Results

### AFF4 regulates *ESR1* gene expression

Previously we reported that bromodomain-containing proteins, BRD3/4, regulate transcription of *ESR1*^16,17^. As reported in literature, the BRD4 protein activates transcription through recruiting the positive transcription elongation factor, P-TEFb, which is composed of two subunits, cyclin T1 and CDK9^18^. Once activated, CDK9 phosphorylates the carboxyl terminal domain (CTD) of RNA polymerase II and promotes transcription elongation. In mammalian cells, the active form of P-TEFb is tightly associated with additional elongation factors ELL1/2/3, EAF1/2, AF9/ENL, as well as AFF4/1 to form the Super Elongation Complex (SEC)^19–22^. Therefore, SEC is also likely involved in transcriptional regulation of *ESR1* expression.

**Fig. 1:**
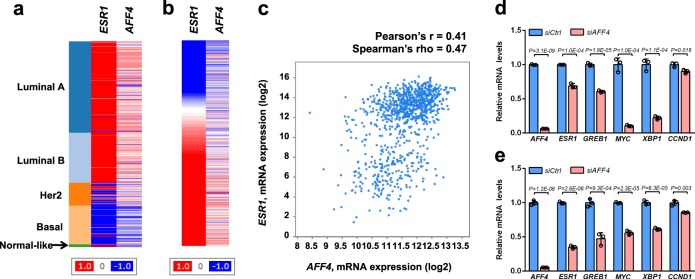
AFF4 preferentially expresses in ER-positive tumors, and its expression positively correlates with ESR1 gene expression. **a**, **b** Heatmaps generated from UCSC genome cancer browser (https://genome-cancer.ucsc.edu), and the TCGA breast invasive carcinoma gene expression dataset has been used for analysis (with Agilent G4502A_07_3 array). In (**a**), the gene expression of ESR1 and AFF4 was arranged by PAM50 subtypes; and in (**b**), AFF4 gene expression was arranged by ESR1 expression in all tumor samples in this cohort. **c** A scatterplot showing the correlation between ESR1 mRNA and AFF4 mRNA based on TCGA breast invasive carcinoma dataset published in Cell 2015, with 971 complete tumors. The plot was generated from cBioPortal for Cancer Genomics website (http://www.cbioportal.org). **d**, **e** siRNA knockdown of AFF4 reduces mRNA levels of ESR1 and its target genes in MCF7 (**d**) and T47D (**e**) breast cancer cells. MCF7 and T47D cells were transfected with non-targeting control siRNA (siCtrl) or AFF4 siRNA (siAFF4), and total RNA were extracted for RT-qPCR 48 h after transfection. Beta-actin has been used to normalize the mRNA levels of all genes measured in the figure. The error bars were shown as SD from biological triplicates, *n* = 3; *P-*values were determined by two-tailed Student’s *t*-test.

We then set to investigate if the SEC plays a role in *ESR1* gene expression. We chose to focus on AFF4, the scaffold protein that interacts with all the subunits in the SEC complex, including P-TEFb, ELLs, and AF9/ENL^23^. We first examined the *AFF4* mRNA levels in different breast cancer PAM50 subtypes based on the TCGA breast invasive carcinoma gene expression dataset. *AFF4* mRNA is highly expressed in luminal A, luminal B, and some Her2-positive tumors, but is absent in the basal subtype of breast tumors, suggesting that the expression of *AFF4* and *ESR1* has a positive correlation (Fig. 1a, b). The correlation of *ESR1* and *AFF4* expression in the TCGA breast invasive carcinoma dataset with 971 completed tumors was calculated. The Pearson’s r was 0.41, and Spearman’s rho was 0.47, indicating a positive correlation (Fig. 1c).

Next, we asked if depletion of AFF4 would reduce *ESR1* expression. We knocked down *AFF4* expression with siRNAs in two luminal A breast cancer cell lines, MCF7 and T47D. Expression levels of *ESR1* mRNA and its target genes were evaluated by RT-qPCR analysis. As shown in Fig. 1d, e, AFF4 ablation decreased *ESR1* expression by more than 50%, and reduced most of the ERα target genes tested in both cell lines, suggesting AFF4 is a regulator of *ESR1* gene expression. We also measured the gene expression of estrogen-responsive genes. Shown in Supplementary Fig. 1, AFF4 was ablated with two different siRNAs in MCF7 cells, and the effects on E2-responsive genes *pS2* and *XBP1* were determined by RT-qPCR. It appears that AFF4 was essential for expression of ER target genes in both hormone-dependent and independent manners.

### AFF4 interacts with histone H3K27ac in vitro

**Fig. 2:**
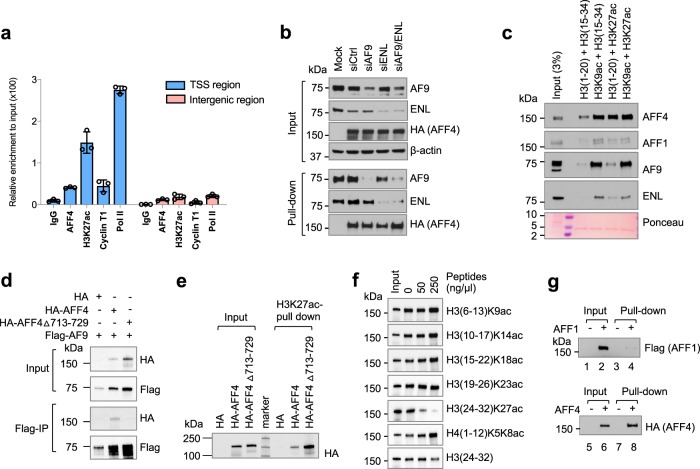
AFF4 interacts with K27-acetylated histone H3 in vitro. **a** ChIP-qPCR was performed in MCF7 cells to determine the recruitment of AFF4, H3K27ac, Cyclin T1 and RNA polymerase II to *ESR1* gene. Primers for TSS region of *ESR1* gene and primers for an intergenic region on 3′ of *ESR1* gene were used for qPCR. The error bars were shown as SD from biological triplicates. **b** H3K27ac peptide pull-down assay to determine the binding of AFF4 in the absence of YEATS protein AF9 or/and ENL. 293 T cells were used for overexpression of AFF4 and knockdown of AF9 and ENL. Biotinylated H3K27ac (aa.23–42) was used as a bait. **c** In vitro histone peptide pull-down assay using HeLa nuclear extract as the input. H3 (1–20) is an unmodified control peptide for H3K9ac, and H3 (15–34) is an unmodified control peptide for H3K27ac. Ponceau staining shows the Streptavidin on Dynabeads M-280. **d** Co-Immunoprecipitation experiment to examine the interaction between AF9 and AFF4 deletion mutant. Amino acids 713–729, a leucine-rich region that mediates the interaction of AFF4 and ENL/AF9, has been deleted in this mutant. **e** H3K27ac peptide pull-down assay to determine the interaction between the AFF4 △713–729 mutant and H3K27ac peptide. **f** Peptide competition experiment to determine selectivity of the interaction between AFF4 and H3K27ac. **g** 293 T cell lysate with overexpressed AFF1 (Flag-tagged) or AFF4 (HA-tagged) was used for pull-down. 10% input was loaded for western blot.

The involvement of AFF4 in regulation of *ESR1* gene expression confirms the importance of the SEC’s role in *ESR1* gene regulation. Previously, we identified the SEC as a H3K27ac-binding complex using peptide pull-down and mass spectrum analysis^24^. Because H3K27ac is a key epigenetic mark that positively correlates with gene activation, this finding suggests that SEC cooperates with histone modifications in controlling *ESR1* gene activity. Indeed, ChIP-qPCR analysis showed that H3K27ac, AFF4, Cyclin T1, and RNA pol II are all enriched in the TSS region of the *ESR1* gene, but absent from the intergenic regions downstream of the *ESR1* gene (Fig. 2a).

We then set out to identify which subunit in the SEC is responsible for H3K27ac binding. AFF4 appeared to be a good candidate because its immunoprecipitation signal was consistently stronger than other SEC subunits as shown in our previous publication^24^. However, two other subunits of the SEC, namely AF9 and ENL, were reported to have H3K27ac-binding activity through their YEATS domains^25,26^. Therefore, we first examined if AFF4 interacts with H3K27ac through AF9 or ENL. After knocking down AF9 and ENL individually or simultaneously, we then examined if AFF4 could still interact with H3K27ac by peptide pull-down assay. As shown in Fig. 2b, the interaction between AFF4 and H3K27ac peptide was not impaired by depletion of AF9 and/or ENL, indicating that AFF4 may directly interact with H3K27ac. This is further supported by an in vitro peptide pull-down assay using HeLa nuclear extract. The H3K27ac peptide efficiently pulls down AFF4, but not AF9 or ENL (Fig. 2c). Instead, AF9 and ENL displayed a strong interaction with the H3K9ac peptide. Moreover, AFF4 has been found to interact with both AF9 and ENL through a leucine-rich sequence^23^. Based on this, we have deleted this region (aa.713–729). This deletion mutant indeed lost the interaction between AFF4 and AF9, as shown by the co-IP assay in Fig. 2d. H3K27ac peptide binding assay showed that the deletion mutant can bind to H3K27ac at least to the same level as the wild-type, further confirming that AF9/ENL is not needed for AFF4 to interact with H3K27ac (Fig. 2e).

In order to examine the selectivity of AFF4 with acetylated histones, various concentrations of acetylated peptides were incubated together with cell lysates that containing overexpressed AFF4, followed by a biotinylated H3K27ac peptide pull-down experiment. As expected, the H3K27ac peptide competed the binding but other peptides did not (Fig. 2f). Interestingly, several acetylated peptides increased AFF4 binding at the highest concentrations. This result suggests that AFF4 interacts with H3K27ac in a form of protein complex, which may contain several other acetylated histone binding proteins. The multiple interactions between acetylated histones at various sites and histone binding proteins will likely stabilize each other, and consequently retain the entire complex on chromatin.

AFF4 belongs to the AFF(AF4/FMR2) family which contains four members: AFF1/AF4, AFF2/FMR2, AFF3/LAF4, and AFF4/AF5q31^27^. These proteins are highly homologous and share similar functions. AFF4 and AFF1 can form heterodimers and both function as the scaffold proteins for the SEC complex^28^. Therefore, we examined if AFF1 can also interact with H3K27ac. Shown in Fig. 2g, the exogenously expressed AFF4 protein, but not its homolog AFF1, could robustly bind to H3K27ac peptide, suggesting that the same family of proteins display different binding ability.

### AFF4 interacts with H3K27ac through its C-terminus

**Fig. 3:**
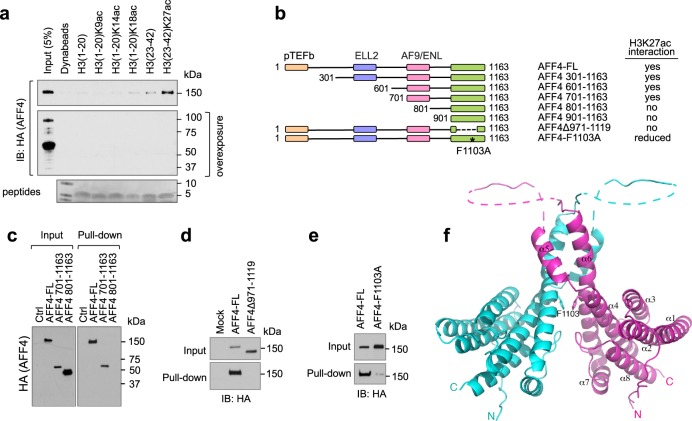
AFF4 interacts with H3K27ac through its C-terminal region. **a** Histone H3K27ac peptide pull-down assay. 293 T cell lysate with overexpressed AFF4 (HA-tagged) was used for pull-down. The blot with degraded forms of AFF4 was exposed for an extended time. Histone peptides used for the pull-down assay were stained with GelCode^TM^ Blue protein stain. **b** A schematic summary of AFF4 deletion mapping to determine the H3K27ac-interacting region. The regions mediating the interaction between AFF4 and other SEC subunits were indicated by different colors. The key results from the peptide pull-down assay were shown in **c**–**e**. In all experiments shown in this figure, 293 T cell lysate with overexpressed AFF4 (HA-tagged) was used for pull-down. **f** Structure of the AFF4 homodimerization domain. A cartoon representation of the AFF4 C-terminal dimerization domain is depicted, with one monomer shown in magenta and another in cyan. The dashed-lines represent disordered loop segments encompassing residues 1033–1040 and 1047–1080. Phe1103 is shown as a stick model.

We next tried to delineate the H3K27ac-interacting domain on AFF4. Interestingly, on an overexposed blot showing degraded AFF4 proteins, we noticed that the H3K27ac peptide failed to pull down the degraded AFF4 protein fragments. Since the expressed AFF4 contains a N-terminal HA-tag, this result suggests that C-terminus of AFF4 protein is essential for its interaction with H3K27ac (Fig. 3a). Therefore, we further performed deletion mapping of AFF4 from its N-terminus while keeping its C-terminus intact (Fig. 3b). Interestingly, deletion of a.a. 701–800 of AFF4 abrogated its binding to H3K27ac peptide, suggesting this 100-amino-acid region is essential to the interaction (Fig. 3c). Additionally, since the flanking sequences of H3K9 and H3K27 are highly similar, and both AF9 and ENL can interact with H3K27ac through the YEATS domain in vitro, we performed a sequence alignment between AFF4 and YEATS domains from AF9 and ENL to see if any homologous domain can be identified (http://multalin.toulouse.inra.fr/multalin/). The alignment suggested residues 971–1117 of AFF4 as a potential homologous region between AFF4 and YEATS domains. Supplementary Fig. 2a shows the alignment of AFF4 C-terminus with YEATS domains. As expected, when we deleted this region from AFF4 protein, this deletion mutant completely lost its interaction with H3K27ac peptide (Fig. 3d). Furthermore, the critical residues identified in the H3K9ac binding cavity appear to be conserved in AFF4. We then made a single amino acid mutation of Phe1103 (F1103A), which corresponds to Phe81 in AF9, one of the key residues responsible for H3K9ac binding in the YEATS domain. As shown in Fig. 3e, this single mutation dramatically decreased the interaction between AFF4 and H3K27ac peptide, further confirming that AFF4 interacts with H3K27ac through its C-terminal region.

### Structure of the C-terminal domain of AFF4

To gain a better understanding of the molecular basis by which AFF4 interacts with H3K27ac, we set out to determine the crystal structure of the C-terminal domain of AFF4. We were unable to crystallize the fragment encompassing residues 701–1163, which is competent for binding H3K27ac in our pull-down experiment. However, we did succeed in crystallizing and solving the structure of an AFF4 fragment encompassing residues 900–1163. Surprisingly, the 2.2 Å structure shows an all α-helical homodimeric structure, instead of the predominantly β-sheet YEATS domain-like structure (Fig. 3f). The ordered portion of the structure, containing residues 902–1160, with the exception of a disordered internal segment from residue 1047 to residue 1080, forms eight α-helices. Helices α5 and α6 are extensively involved in homodimerization. Phe1103, the residue identified to be important for H3K27ac interaction, is located in the middle of α6 and involved in dimerization via stacking with the same residue from another molecule. In consistent with the structural result, AFF4 dimerization was abolished by a rather large deletion of residues 971–1117, but was not affected by the single mutation F1103A in cells (Supplementary Fig. 2c). A similar structure is obtained in an independent recent study^29^.

Our structural result contrasts the expectation of a YEATS domain suggested by sequence alignment, and precludes direct participation of Phe1103 in H3K27ac interaction in the present form. However, a caveat is that the a.a. 901–1163 domain we crystallized is not competent for H3K27ac binding in our pull-down experiment (Supplementary Fig. 2b). Moreover, this domain lost the ability to dimerize with the full-length AFF4 in cells (Supplementary Fig. 2d). Various scenarios of H3K27ac binding, which we will discuss in detail later, are possible with the inclusion of the region encompassing residues 701–800, as in the pull-down experiments. To reconcile the seemingly divergent conclusions from our in vitro binding and structural results, we digress to seek evidences in cells.

### AFF4 associates with H3K27ac on genome

**Fig. 4:**
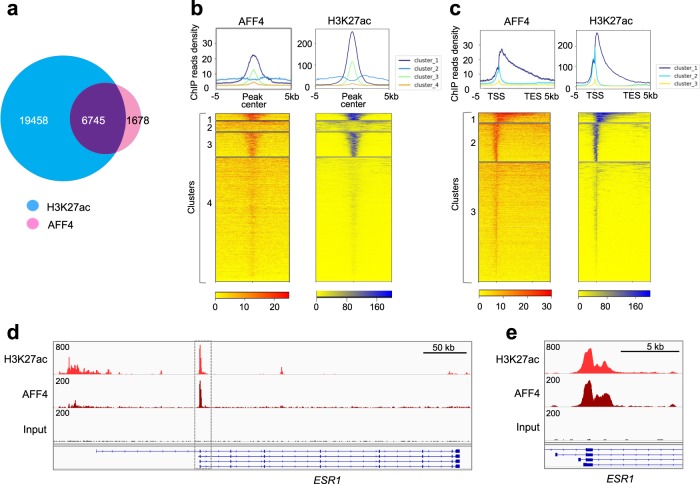
AFF4 colocalizes with H3K27ac on chromatin in MCF7 cells. **a** Venn diagram depicting common and unique binding sites among AFF4 and H3K27ac cistromes in MCF7 cells. **b**, **c** Global positive correlation between AFF4 and H3K27ac cistromes. Aggregation plots (top) and corresponding heatmaps (bottom) of AFF4 and H3K27ac cistromes in MCF7 cells after hierarchical clustering. Binding sites are either aligned relative to the AFF4/H3K27Ac peak center (**b**) or relative to the transcription start site (TSS) and transcription termination site (TES) of AFF4/ H3K27ac -bound genes (**c**). **d** IGV tracks showing the colocalization of AFF4 and H3K27ac peaks on transcription start site (TSS) of *ESR1* gene. **e** A magnified figure of the rectangle shown in **d**.

Next, we investigated whether there is a functional interaction between AFF4 and H3K27ac in the cells. We performed H3K27ac and AFF4 ChIP-seq in MCF7 cells. In this assay, 80% of AFF4 genomic binding sites overlap with H3K27ac-enriched sites (Fig. 4a). Moreover, hierarchical clustering of AFF4/H3K27ac-binding peaks suggests that AFF4 and H3K27ac are highly enriched in the TSS/5′-UTR region, with a spreading towards the gene body region (Fig. 4b, c). The Pearson correlation coefficient between AFF4 and H3K27ac-binding peaks has been calculated to be 0.59, supporting their functional and physical association on chromatin. When we examined the *ESR1* gene locus, we found a remarkable peak in the TSS region where both H3K27ac and AFF4 were highly enriched, indicating that AFF4 is recruited to the *ESR1* gene with H3K27ac modification (Fig. 4d, e).

**Fig. 5:**
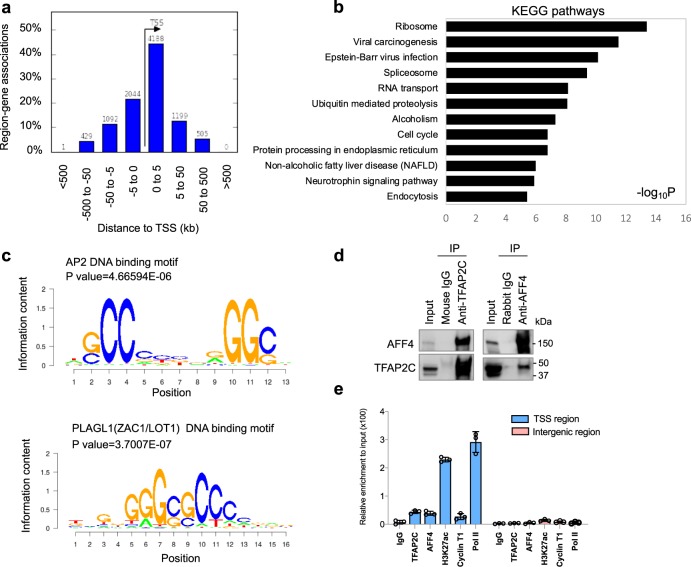
Analysis of the AFF4/H3K27ac cistrome in MCF7 cells. **a** Distribution of the distances between AFF4 binding sites to transcription start site (TSS) on the AFF4/H3K27ac co-binding peaks. The *Y*-axis displays the number of genes associated with each binding site. **b** Gene ontology of top enriched KEGG pathways on genes associated with these peaks. **c** Top enriched motifs associated with AFF4/H3K27ac co-binding peaks. **d** Co-immunoprecipitation (co-IP) experiment to determine the interaction between AFF4 and TFAP2C. 293 T cells were transiently transfected with expression vectors for TFAP2C and AFF4. Reciprocal IP was performed 48 hours later. Input, 3%. **e** ChIP-qPCR to determine the recruitment of TFAP2C, AFF4, H3K27ac, Cyclin T1, and RNA polymerase II to *ESR1* gene. Primers for TSS region of *ESR1* gene and primers for an intergenic region on 3′ of *ESR1* gene were used for qPCR. The error bars were shown as SD from biological triplicates, *n* = 3.

Next, we performed a genome-wide analysis of AFF4/H3K27ac co-binding sites. Similar to the binding pattern on *ESR1* gene, a majority of the ChIP-seq peaks are located on the genomic sequences within 5 kb upstream/downstream of TSS and are particularly enriched in the 5′-UTR regions (Fig. 5a). Supplementary Fig. 3 shows the IGV tracks of AFF4 and H3K27ac peaks on their target genes including *GATA3, FOXA1, DDIT4, XBP1, MYC, NEAT1*, and *FAM84B*. This observation is in consistent with the function of AFF4 as a transcriptional elongation factor. We further performed KEGG pathway enrichment analysis on AFF4/H3K27ac-associated genes. Figure 5b shows the pathways that are most statistically significant. Among them, ribosome, spliceosome, and RNA transport pathways are involved in protein synthesis. Regulation of genes in these anabolic pathways by AFF4 is consistent with the role of ERα in promoting cell proliferation.

### AFF4 associates with TFAP2C

The ChIP-seq results also allowed us to identify transcription factor binding motifs that associate with AFF4/H3K27ac-binding sites. We identified consensus binding motifs for two groups of transcription factors: AP2 and PLAGL1 (ZAC1/LOT1) (Fig. 5c). Whereas no evidence indicates that PLAGL1 plays a role in estrogen signaling, a few studies reported that transcription factor AP-2 gamma (TFAP2C) regulates the expression of *ESR1*^30–32^. In consistent with previous results, we observed a substantial enrichment of H3K27ac and AFF4 in the 5′-UTR of *TFAP2C*, suggesting that *TFAP2C* is expressed in MCF7 cells (Supplementary Fig. 4a). In contrast, H3K27ac is barely detectable on the *PLAGL1* gene (Supplementary Fig. 4b), implying that *PLAGL1* is not expressed in MCF7 cells. Therefore, we focused our study on TFAP2C.

The TFAP2 (AP-2) family of transcription factors is composed of five members: TFAP2A, TFAP2B, TFAP2C, TFAP2D, and TFAP2E^33^. AP2 dimers recognize palindromic GC-rich DNA response elements with the consensus sequence 5′-GCCNNNGGC-3′^34^. Among the five proteins, only TFAP2A and TFAP2C are expressed in MCF7 cells. It has been reported that TFAP2C regulates *ESR1* gene expression, but not TFAP2A^32^. Therefore, the interaction between AFF4 and TFAP2C was examined. The reciprocal co-immunoprecipitation result confirms their interaction (Fig. 5d). And as expected, TFAP2C binds to the TSS region of *ESR1* gene, but is absent from the intergenic region downstream of *ESR1* gene (Fig. 5e), consistent with the chromatin localization of AFF4.

### AFF4 ablation suppresses breast cancer cell growth

**Fig. 6:**
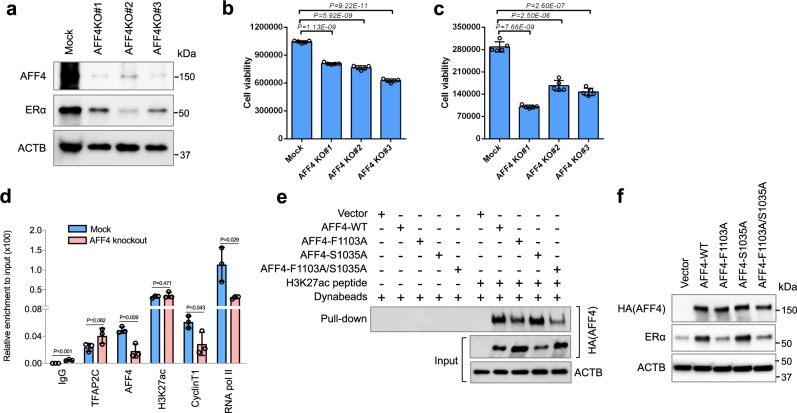
AFF4 ablation in MCF7 cells inhibits ERα expression and cell growth. **a** AFF4 was knocked out in MCF7 cells using CRISPR/Cas9 technology. Cell lysate from a control and three different AFF4-knockout clones was extracted and subjected to western blot analysis to determine the expression levels of AFF4 and ERα. **b** Knockout of AFF4 reduced the growth of MCF7 cells in culture medium containing regular FBS. **c** Knockout of AFF4 reduced the growth of MCF7 cells in culture medium with charcoal-stripped FBS. **d** AFF4 knockout reduced the recruitment of Cyclin T1 and RNA polymerase II to *ESR1* gene. Chromatin prepared from the control or AFF4-knockout MCF7 cells was used for ChIP-qPCR. Primers for TSS region of *ESR1* gene were used for qPCR. Cells were cultured in medium containing charcoal-stripped FBS. **e** Amino acid F1103 of AFF4 is critical for AFF4 to interact with H3K27ac. 293 T cells were transfected with empty vector or different AFF4 mutants, and the cell lysate was collected for H3K27ac peptide pull-down assay. **f** Amino acid F1103 of AFF4 is essential for ERα expression. AFF4-knockout MCF7 cells were transfected with empty vector or different AFF4 mutants, and western blot assay was performed to determine the protein levels of AFF4 and ERα. Cells were cultured in medium containing charcoal-stripped FBS. The error bars were shown as SD; *n* = 5 (**b**, **c**) or 3 (**d**) biological replicates; *P*-value was determined by two-tailed Student’s *t*-test.

Due to the importance of ERα in breast cancer cell growth, we further examined the effect of AFF4 ablation on cell growth. To this end, we depleted AFF4 from MCF7 cells and T47D cells by CRISPR/Cas9 technology, and the growth of these cells was examined in culture media containing regular FBS or charcoal-stripped serum. Figure 6a lists three MCF7 AFF4-knockout cell lines that showed successful ablation of AFF4. As expected, ERα levels were reduced in AFF4 KO lines. Same results were obtained from T47D cells (Supplementary Fig. 5a). In consistence with previous report in which knockdown of TFAP2C decreased the number of cells in S phase and delayed the growth of xenograft tumor in both E2− and E2 + conditions, we observed a reduction of cell viability in all six AFF4-knockout lines (Fig. 6b, c, Supplementary Fig. 5b, c). Interestingly, the extent of growth inhibition was particularly compelling when MCF-7 cells were cultured in media with charcoal-stripped sera, indicating that AFF4 is crucial for the growth of ER-positive breast cancer cells, and ablation of AFF4 may sensitize breast cancer cells to estrogen-deprived therapy.

We then examined if there was any change in transcription factor/coactivator recruitment upon AFF4 ablation. Using ChIP-qPCR, Cyclin T1 and RNA pol II recruitment was revealed to be markedly reduced on the *ESR1* gene, suggesting that the loss of AFF4 decreased the recruitment of P-TEFb and transcription preinitiation complex (Fig. 6d).

Finally, in order to evaluate the impact of interaction between AFF4 and H3K27ac on *ESR1* gene expression, we performed a rescue experiment in MCF7 AFF4-KO cell lines. Previously when we made H3K27ac-binding mutants based on the homology between AFF4 and YEATS domains, we also mutated Ser1035 to Alanine, which correlates to Ser58 of AF9 that locates within the H3K9ac binding pocket. Interestingly, the single mutation of S1035A did not disrupt its H3K27ac-binding activity, but the double mutant F1103A/S1035A displayed the lowest H3K27ac-binding activity among all four AFF4 proteins tested (Fig. 6e). We thus included these two additional mutants in the rescue experiment. Shown in Fig. 6f, re-expression of wild-type AFF4 or AFF4-S1035A in AFF4-KO cells restored the ERα level, but not the AFF4-F1103A or the double mutant, suggesting that the function of AFF4 on transcription is largely dependent on its H3K27ac-binding activity.

## Discussion

**Fig. 7:**
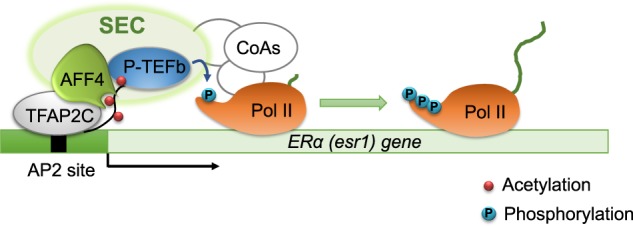
Working model. H3K27ac signals the transition from transcriptional initiation to elongation. The SEC is recruited to TSS of *ESR1* gene through the interaction between AFF4 and H3K27ac. The interaction between TFAP2C and AFF4 further stabilizes the SEC, and facilitates the recruitment of additional coactivators, mediators, and RNA polymerase II. P-TEFb phosphorylates CTD of RNA pol II and allows the pause-release of RNA pol II for transcriptional elongation.

Endocrine therapy has been very successful in the treatment of ER-positive breast cancers. But endocrine resistance often develops, and currently there is no effective therapeutic intervention for endocrine-resistant breast cancer. In the resistant tumors, ERα continues to be an essential transcription factor for tumor growth. Therefore, diminishing *ESR1* gene expression becomes a promising treatment strategy. The current study reveals how *ESR1* gene expression is regulated at the level of transcriptional elongation. Based on our findings, we propose a working hypothesis as shown in Fig. 7. The transcription of *ESR1* gene can be modulated at both initiation and elongation stages, and acetylation of histone H3 at K27 signals the transition from transcription initiation to elongation by recruiting the SEC complex. Importantly, AFF4, the scaffold subunit of the SEC, is largely responsible for this histone mark recognition activity. Depletion of AFF4 by knockdown or knockout reduces the expression of *ESR1* and its target genes, and AFF4 mutants that lacks H3K27ac interaction fail to rescue *ESR1* expression.

In our pull-down experiments, we find that the AFF4 region encompassing residues 701–1163 interacts with H3K27ac. Lacking regions spanning residues 701–800 or 901–1163 compromises the ability for AFF4 to interact with H3K27ac. Our structural result reveals that the 901–1163 region of AFF4 is a dimerization module, instead of a complete H3K27ac-interacting domain. Several scenarios may account for our observations. First, it is possible that in the context of full-length protein or presence of the residue 701–800 region, the C-terminal homodimerization domain we observed in the structure may be engaged in interaction with other regions of the protein, forming a new tertiary structure or quaternary configuration suitable for binding H3K27ac. In support of this possibility, our pull-down experiment shows that deletion of residues 701–800 affect self-association of AFF4 in the cell (Supplementary Fig. 2d). Another scenario is that the detected interaction may be mediated by a yet unidentified factor stably associated with AFF4, as we detected H3K27ac binding by IP and pull-down assays from cell lysate. This factor is not AF9 or ENL, which are known proteins associated with residue 701–800 region of AFF4, as depletion of AF9 or/and ENL does not affect AFF4 binding with H3K27ac (Fig. 2b, e). Regardless of detailed molecular mechanisms of H3K27ac binding by AFF4, our data clearly indicate that AFF4 mediates H3K27ac association in cells.

In MCF7 cells, genome-wide ChIP-seq analysis reveals that 80% of AFF4 genomic binding peaks overlap with H3K27ac peaks. Interestingly, 90% of AFF4 binding peaks are located within ±50 kb distance to TSS of target genes, and 66% are located within ±5 kb distance to TSS, suggesting that AFF4 is a TSS-related transcriptional coregulator (Fig. 5a). In contrast, only 26% of H3K27ac peaks overlap with AFF4 peaks. One possibility is that histones are integral structural proteins of chromatin and abundant, and therefore their ChIP-seq signals are strong. Consequently, more peaks would be generated by H3K27ac ChIP-seq. On the other hand, similar to many H3K27ac ChIP-seq results published before, we also found that H3K27ac was enriched at both TSS regions and enhancers, with similar signal intensity (Supplementary Fig. 6). This observation suggests that AFF4 colocalizes with H3K27ac only at genomic regions close to TSS and 5′-UTR, rather than on enhancers (Fig. 4c). It is likely that additional transcription factors, such as TFAP2C, help to secure the recruitment of AFF4 on the TSS region. Importantly, a previous study reported that TFAP2B interacts with GAS41^35^, a YEATS domain-containing protein that preferentially binds to H3K27ac and H3K18ac^36,37^, suggesting the interaction between TFAP2 family of transcription factors and acetylated histone-interacting proteins might serve as a general mechanism to facilitate transcriptional progression from initiation to elongation.

Moreover, our study indicates that histone H3K27ac can cooperate with transcription factors to determine the specificity of gene activation. The SEC belongs to a large family of SEC-like complexes, including SEC, SEC-L2, and SEC-L3, etc^22^. These complexes were classified by different scaffold proteins, and they all share the common subunits such as P-TEFb and acetylated histone binding proteins AF9/ENL. Although AFF1 and AFF4 were initially identified as heterodimers and both were components of SEC in pull-down assays, they have been reported to regulate discrete subsets of target genes, and display very different affinity with their associated transcription factors^38–40^. Therefore, AFF1 and AFF4 may form individual SECs and the gene-specificity of each SEC complex is likely determined by its associated proteins. For instance, in comparison with AFF4, AFF1 has much higher affinity binding with HIV transcription factor Tat because of a single amino acid variance in the N-terminal binding sites^40^. In this study, we found that AFF4 associates with H3K27ac, but AFF1 does not. Therefore, the identification of different properties between the highly similar scaffold proteins can assist the development of strategies targeting specific SEC complexes.

## Methods

### Cell culture and transfection

MCF7, T47D, and HEK293T cells were obtained from Tissue and Cell Culture Core at Baylor College of Medicine. Cells were maintained in RPMI 1640 medium (MCF7 and T47D) or Dulbecco’s Modified Eagle Medium (HEK293T) supplemented with 10% FBS. Phenol red-free medium containing 10% charcoal-dextran-stripped FBS was used for estrogen-deprived culture. Possible mycoplasma contamination is routinely monitored once every 6 months in the lab. TransIT^®^-LT1 transfection reagent (Mirus) was used for transient transfection experiments with plasmid DNA. AFF4 SMARTpool siGENOME siRNA were purchased from Dharmacon, and was transfected to cells with Lipofectamin RNAiMAX reagent in a final concentration of 20 nM. The mammalian expression vector of TFAP2C (pCS6, BC051829) was purchased from TransOMIC Technologies. pRK5M-AFF4 expression vector was generously provided by Dr. Qiang Zhou (UC Berkeley). AFF4 mutants (F1103A, S1035A, and F1103A/S1035A) were generated using Q5 Site-directed mutagenesis kit (NEB). To re-express AFF4 in AFF4-knockout MCF7 cells, a single nucleotide G to C mutation was introduced at nucleotide 1356 (NM_014423) in the pRK5m-AFF4 vector. This mutation disrupts the so-called PAM sequence (the last three nucleotides 5′-TGG-3′) of AFF4 sgRNA, without altering the AFF4 protein coding sequence.

### CRISPR–Cas9-mediated AFF4 knockout

Lentivirus-based hPGK-puro-2A-tBFP vector containing AFF4 sgRNA oligonucleotide (sequence: 5′-TCTGGTGATGTGAGCTGTGTGG-3′) was purchased from SIGMA-ALDRICH. The virus was packaged following the manufacturer’s instructions. To obtain the AFF4-knockout breast cancer cell line, MCF7 and T47D cells were transduced with Cas9 expressing lentiviruses and selected by 10 µg/ml blasticidin for 1 week, followed by a transduction with a lentivirus containing AFF4 sgRNA. MCF7 and T47D cells were further selected with 2.5 µg/ml puromycin for 2 weeks. Individual AFF4-knockout clones were selected for MCF7 and T47D cells and verified by western blot analysis.

### Histone peptide pull-down

Biotinylated histone peptides were purchased from JPT Peptides Inc. and Epicypher. For each pull-down analysis, 0.25 μg of peptide was immobilized onto 25 μl Dynabeads M-280 streptavidin (Life Technology, Inc), followed by incubation with 1 mg cell lysate at 4 °C for 3 h. After binding, the beads were washed three times with wash buffer BC150 (20 mM Tris–HCl pH 7.9, 10% glycerol, 0.2 mM EDTA, 150 mM KCl) and mixed with 2× SDS sample buffer before subject to SDS-PAGE separation and western blot analysis. The peptides used for competition experiment shown in Fig. 2f were synthesized from GenScript. The peptide sequences are: H3(6-13)K9ac: QTARK(ac)STGG; H3(10-17)K14ac: STGGK(ac)APR; H3(15-22)K18ac: APRK(ac)QLAT; H3(19-26)K23ac: QLATK(ac)AAR; H3(24-32)K27ac: AARK(ac)SAPAT; H3(24-32): AARKSAPAT; H4(1-12)K5K8ac: MSGRGK(ac)GGK(ac)GLG.

### Co-immunoprecipitation (Co-IP) and western blot analysis

Co-IP experiments were performed to determine the interaction between AFF4 and TFAP2C proteins. Two days after a transient transfection, 293 T cells were washed with ice-cold phosphate-buffered saline, then lysed with lysis buffer (50 mM Tris, 100 mM NaCl, 0.1% NP-40, 50 mM NaF, 1 mM dithiothreitol, 1 mM phenylmethylsulfonyl fluoride, and 1 × protease inhibitor cocktail). Two microgram antibody and 25 µl protein G beads were used for immunoprecipitation at 4 degree for 4 h. After three washes with lysis buffer, the immunoprecipitated proteins were separated by SDS-PAGE and analyzed by western blot. The antibodies used in co-IP and Western blot were anti-TFAP2C (Santa Cruz, sc-53162), anti-HA antibody (Roche), anti-AFF4 (Bethyl Labs, A302–538A), anti-β-actin (Sigma, A2228), and anti-ERα (Abcam, ab108398).

### Chromatin IP (ChIP)

The ChIP-seq analysis of H3K27ac and AFF4 was performed by Active Motif, Inc (Carlsbad, CA). Specifically, cells were fixed with 1% formaldehyde for 15 min, followed by incubation with a stop solution containing 0.125 M of Glycine. After washing with ice-cold PBS, the fixed cells were snap-frozen, and subject to ChIP-seq at Active Motif. The 75-nt sequence reads generated by Illumina sequencing are mapped to the human genome using the BWA algorithm with default settings. Peak calling was performed using MACS^41^. The hierarchical clustering was performed in Cistrome Analysis Pipeline (http://cistrome.org/ap/). The Region-gene association graph was generated using Genomic Region Enrichment Annotation Tool (GREAT, http://great.stanford.edu/public/html/). The gene ontology analysis was performed using DAVID Bioinformatics resources (https://david.ncifcrf.gov).

For ChIP-qPCR assay, we used the ChIP-IT Express kit (Active Motif) following manufacturer’s protocol. The antibodies used in ChIP assay were rabbit IgG (Santa Cruz Biotechnology, sc-2027), mouse IgG (Santa Cruz Biotechnology, sc-2025), anti-H3K27ac (Abcam, ab4729), anti-AFF4 (Bethyl Labs, A302–538A), anti-RNA polymerase II (Abcam, ab817), and anti-Cylin T1 (Santa Cruz Biotechnology, sc-8127). These antibodies are all ChIP grade and have been validated. The ChIP-qPCR primers for amplification of AFF4 binding site on TSS region of *ESR1* gene are: Forward,5′-TGGACAGCAGCAAGCC-3′; Reverse, 5′- CGGAGACACGCTGTTGAG -3′. The primers for amplification of intergenic region downstream of *ESR1* gene are: Forward, 5′- AGAACCCCAAATGGCAGTC-3′; Reverse, 5′- CAGAATGGGCATCCTCTTTG-3′.

### RNA isolation and reverse transcription-qPCR (RT-qPCR) analysis

Total RNA was extracted with RNeasy Mini Kit (Qiagen). To measure the relative mRNA levels, RT-qPCR was performed in an Applied Biosystems7500 fast real-time PCR system (Applied Biosystems, Foster City, CA). The primers were synthesized according to the published primer sequences found in PrimerBank (https://pga.mgh.harvard.edu/primerbank/), or designed with the ProbeFinder program from Roche (http://www.universlprobelibrary.com). Specifically, the primers for amplification of ERα mRNA: Forward, 5′-ATCCACCTGATGGCCAAG-3′; Reverse, 5′-GCTCCATGCCTTTGTTACTCA-3′. GREB1: Forward, 5′-TTCGGCTCACAGAAGTGGAT-3′; Reverse, 5′-GCTGGAGATAATGCCAGTCAG-3′; C-MYC: Forward, 5′-CACCAGCAGCGACTCTGA-3′; Reverse, 5′-ACTCTGACCTTTTGCCAGGA-3′. XBP1: Forward, 5′-CCCTCCAGAACATCTCCCCAT-3′; Reverse, 5′- ACATGACTGGGTCCAAGTTGT-3′. CCND1: Forward, 5′- GCTGTGCATCTACACCGACA-3′; Reverse, 5′-TTGAGCTTGTTCACCAGGAG-3′. The SensiFast SYBR one-step Kit (Bioline) was used for RT-qPCR analysis.

### Cell growth assay

Breast cancer cells were seeded at a density of 2 × 10^3^ cells per well in flat-bottomed 96-well plates (day 0) and grew for 3 days. CellTiter-Glo® Luminescent Cell Viability Assay (Promega) was used to measure cell viability at day 0 and day 3 following the manufacturer’s instructions. After 10 min of incubation, the cell viability was determined by measuring the luminescence using the Synergy™ neo2 multi-mode reader (BioTek).

### Protein expression, purification, and crystallization

Human AFF4 fragment encompassing residues 900–1163 was cloned into a pET28a-smt vector for expression as an N-terminal his-sumo-tagged fusion protein in the E. coli BL21(DE3) codon-plus RIL strain. Production of the AFF4 protein was induced with 0.25 mM IPTG at 16 °C for 20 h. The cells were then harvested, and lysed in a buffer containing 500 mM NaCl, 20 mM Tris-HCl (pH 8.0), 5 mM Imidazole and 1 mM PMSF. The fusion protein was first purified with Ni-chelating resins, followed by cleavage of the his-sumo tag with the sumo protease. The AFF4 protein was further purified through ion-change (HiTrap SP, GE Healthcare) and size-exclusion (Superdex 75 16/60, GE Healthcare) column chromatography. Peak fractions of the protein sample were analyzed by SDS-PAGE and high-purity fractions were pooled and concentrated to ~19 mg/ml in a buffer containing 100 mM NaCl, 20 mM Tris-HCl (pH 8.0) and 1 mM DTT. Selenomethionyl derivative of the AFF4 protein was obtained following the same protocol, except that the E. coli culture was grown in the M9 medium supplemented with selenomethioine.

Crystal screens were carried out at 16 °C using the sitting-drop vapor diffusion method. Crystals of AFF4 grew in a number of conditions with various inorganic salts, such as 1.6–2.0 M (NH_4_)_2_SO_4_, or 0.4–1.6 M NaH_2_PO_4_, K_2_HPO_4_, and with a variety of buffers, such as MES (pH 6.5), HEPES (pH 7.5), CHES (pH 9.5), and CAPS (pH 10.5). The crystal used for data collection was obtained in 2.2 M NaH_2_PO_4_, 0.4 M K_2_HPO_4_, and 0.1 M MES (pH 6.6).

### X-ray crystallography

Before flash-frozen in liquid nitrogen, the crystals were transferred into a reservoir solution drop supplemented with 20% (v/v) glycerol as cryo-protectant. X-ray diffraction data were collected at the beamline BL17U of Shanghai Synchrotron Radiation Facility (SSRF), and processed using the XDS software package. The phase was solved by the single-wavelength anomalous dispersion method using Phenix.autosol. Model building and refinement were performed with Coot and PHENIX.refine, respectively. Statistics for structural analysis are shown in Supplementary Table 1.

### Statistics and reproducibility

Values are expressed as the mean ± standard deviation containing a specified number of replicates. Statistical significance of differences between groups was determined by two-tailed Student *t*-test, and the *P*-values were shown as exact values whenever suitable. Details of the number of biological replicates and the assays are given in each figure legends.

### Reporting summary

Further information on research design is available in the Nature Research Reporting Summary linked to this article.

## Supplementary information

Description of Additional Supplementary Files

Supplementary Information

Supplementary Data 1

Supplementary Data 2

Supplementary Data 3

Supplementary Data 4

Reporting Summary

## Data Availability

The coordinates and X-ray diffraction data have been deposited in PDB with dataset ID: D_1300013315 and PDB ID: 6KN5. The ChIP-seq data are available through GEO with access number GSE144036. Source data of gel and blot images are included in Supplementary Data 1; source data underlying the gene expression results are included in Supplementary Data 2; source data underlying the ChIP assays are included in Supplementary Data 3; and source data underlying the cell growth assay are included in Supplementary Data 4. All other relevant data are available from the corresponding author on reasonable request.
