# Supplementary material for: Acetylation of histone H3K27 signals the transcriptional elongation for estrogen receptor alpha
Source: Commun Biol. 2020 Apr 7;3:165. doi: 10.1038/s42003-020-0898-0 (PMC7138820; doi:10.1038/s42003-020-0898-0)
Supplement: Supplementary file 1 — Description of Additional Supplementary Files [file 42003_2020_898_MOESM1_ESM.pdf]

Description of Additional Supplementary Items

File name: Supplementary Data 1
Description: This file contains all source data of gel and blot figures presented in the main and supplementary figures.

File name: Supplementary Data 2
Description: This file contains all source data underlying the gene expression results and the exact p values in Excel format. This includes results shown in Fig. 1d, Fig. 1e, and Fig. S1.

File name: Supplementary Data 3
Description: This file contains all source data underlying the ChIP-qPCR results in Excel format. This includes results shown in Fig. 2a, Fig. 5e, and Fig. 6d.

File name: Supplementary Data 4
Description: This file contains all source data underlying the cell growth results in Excel format. This includes results shown in Fig. 6b, Fig. 6c, Fig. S5b and Fig. S5c.
