# Supplementary material for: Acetylation of histone H3K27 signals the transcriptional elongation for estrogen receptor alpha
Source: Commun Biol. 2020 Apr 7;3:165. doi: 10.1038/s42003-020-0898-0 (PMC7138820; doi:10.1038/s42003-020-0898-0)
Supplement: Supplementary file 2 — Supplementary Information [file 42003_2020_898_MOESM2_ESM.pdf]

Supplementary Information

Supplementary Table 1. Statistics for crystallographic analysis.

Data collection and refinement statistics.

Data Sets	Se-derivative	Native
**Data collection**		
Space group	I $2_1 2_1 2_1$	I $2_1 2_1 2_1$
Cell dimensions		
a, b, c (Å)	40.82, 71.73, 186.97	41.06, 71.12, 187.42
α, β, γ (°)	90, 90, 90	90, 90, 90
Resolution (Å)	46.74-3.20 (3.42-3.20) *	46.94-2.20 (2.27-2.20)*
R_{merge}	0.113 (0.273)	0.069 (0.946)
Mean I/σ (I)	27.70 (12.20)	21.80 (3.00)
Completeness (%)	100.00 (100.00)	100.00 (100.00)
Redundancy	13.90 (14.30)	12.80 (12.90)
**Refinement**		
R_{work}/R_{free} (%)		22.96/ 27.75
R.m.s. deviations		
Bond lengths (Å)		0.003
Bond angles (°)		0.763
No. reflections		14444
No. atoms		
Protein		1836
Average B factors ($Å^2$)		
Protein		68.11
Ramachandran plot (%)		
Favored		95.93
Allowed		4.07
Outliers		0.00

*Values in parentheses refer to the highest resolution shell.

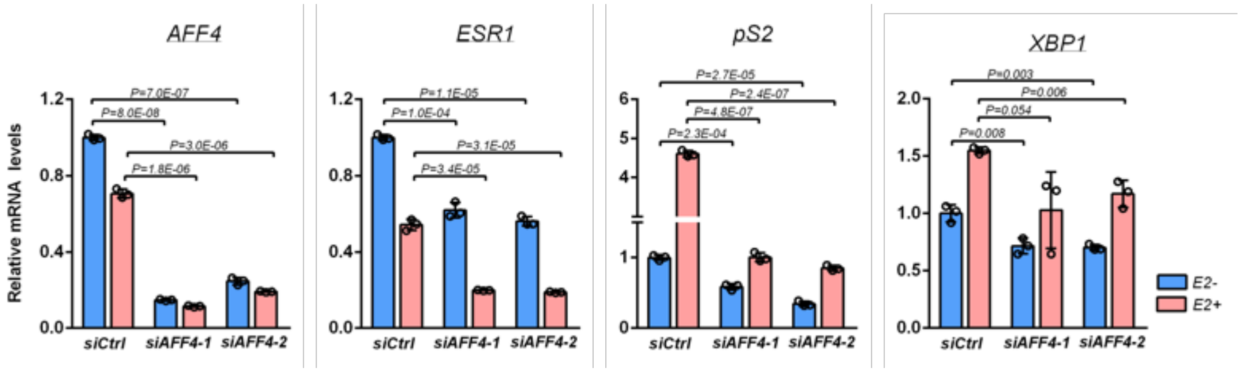

Supplementary Figure 1. siRNA knockdown of AFF4 reduces mRNA level of ESR1 and its target genes independent of E2. The error bars were shown as SD, n=3 biological replicates. *P* values were determined by two-tailed Student's *t*-test.

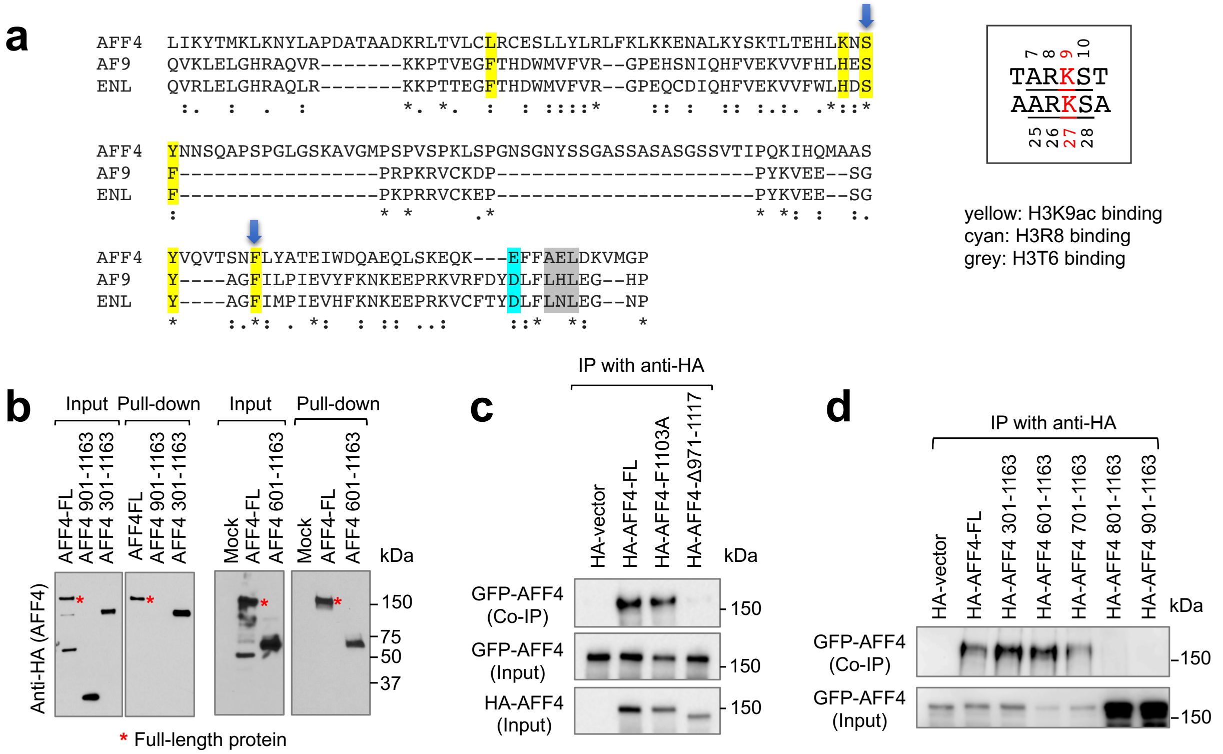

Supplementary Figure 2. AFF4 interacts H3K27ac through its C-terminal region. **(a)** Sequence alignment of AFF4 with YEATS domain of AF9 and ENL by Multalin. The residues highlighted in yellow are critical residues previously identified for H3K9ac binding. The blue arrows refer to two point-mutations made in this study. The sequences shown in the square are histone H3 residues 6-11 and residues 24-29. **(b)** The interaction between H3K27ac peptide and AFF4 deletion mutants. The AFF4 mutants were expressed in 293T cells, and the lysates were collected for an in vitro peptide pull-down analysis. The red asterisks label the full-length AFF4. **(c-d)** Dimerization of GFP-AFF4 (full-length) with HA-AFF4 mutants. IP antibody: anti-HA; Blot antibody: anti-GFP.

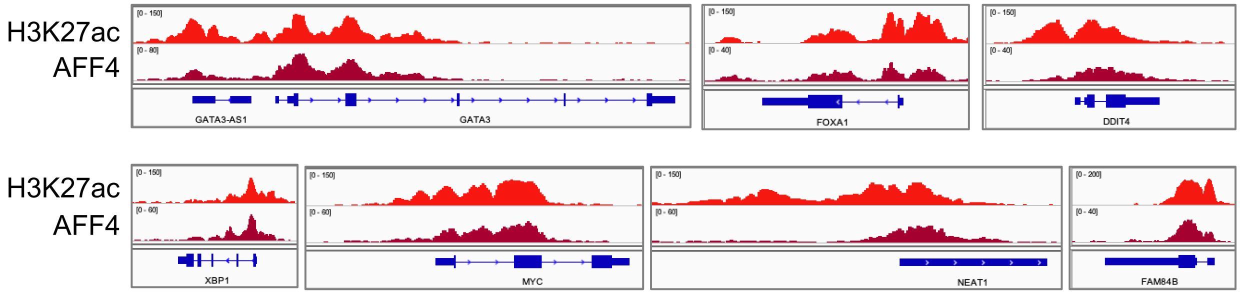

Supplementary Figure 3. IGV tracks showing the co-localization of AFF4 and H3K27ac peaks on their target genes including *GATA3, FOXA1, DDIT4, XBP1, MYC, NEAT1, and FAM84B*.

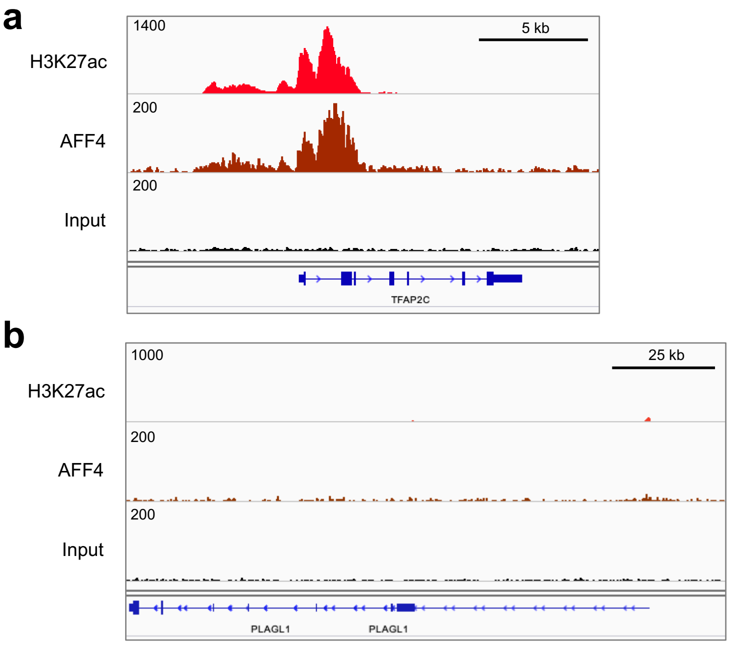

Supplementary Figure 4. IGV tracks showing the enrichment of AFF4 and H3K27ac on transcription start site (TSS) of *TFAP2C* **(a)** and *PLAGL1* **(b)** genes.

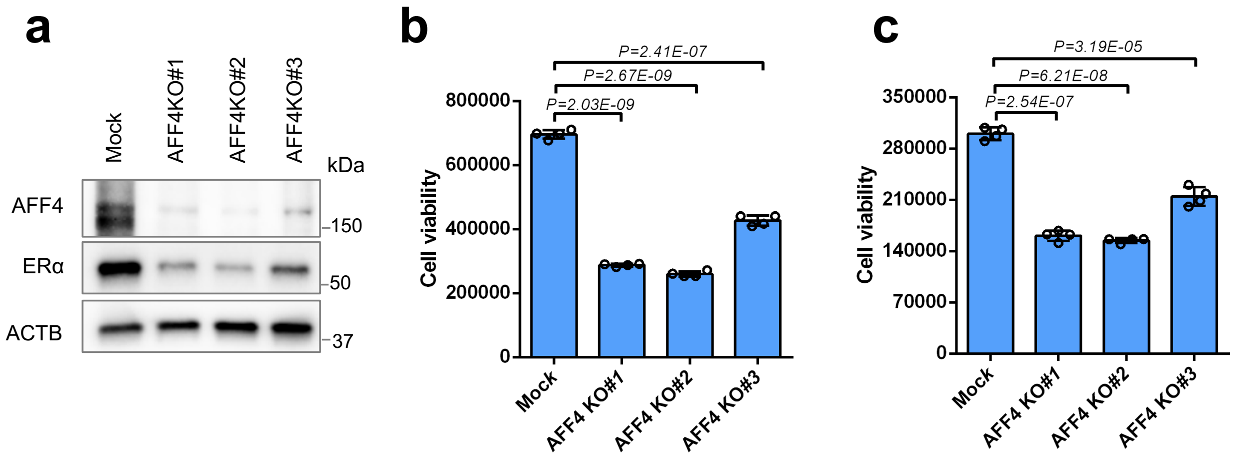

Supplementary Figure 5. AFF4 ablation in T47D cells inhibits ERα expression and cell growth. (a) AFF4 was knocked out in T47D cells using CRISPR/Cas9 technology. Cell lysate from a control and three different AFF4-knockout clones was extracted and subjected to western blot analysis to determine the expression levels of AFF4 and ERα. **(b)** Knockout of AFF4 reduced the growth of T47D cells in culture medium containing regular FBS. **(c)** Knockout of AFF4 reduced the growth of T47D cells in culture medium with charcoal-stripped FBS. In **b** and **c**, the error bars were shown as SD; n=4 biological replicates; *P* value was determined by two-tailed Student's *t*-test.

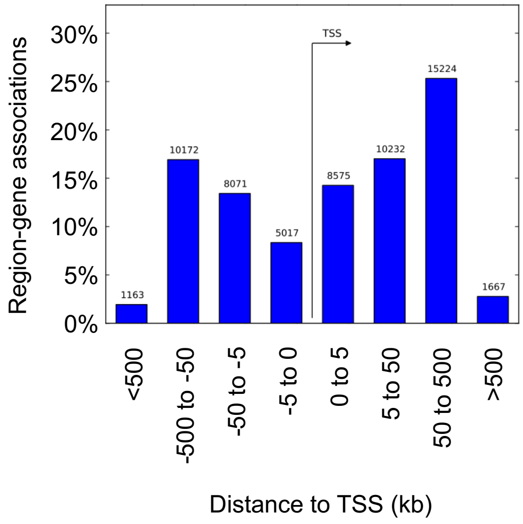

Supplementary Figure 6. Distribution of the distances between H3K27ac binding sites to transcription start site (TSS). The Y-axis displays the number of genes associated with each binding site.
