# Supplementary figures and images for: Acetylation of histone H3K27 signals the transcriptional elongation for estrogen receptor alpha
Source: Commun Biol. 2020 Apr 7;3:165. doi: 10.1038/s42003-020-0898-0 (PMC7138820; doi:10.1038/s42003-020-0898-0)

**Fig.2b**

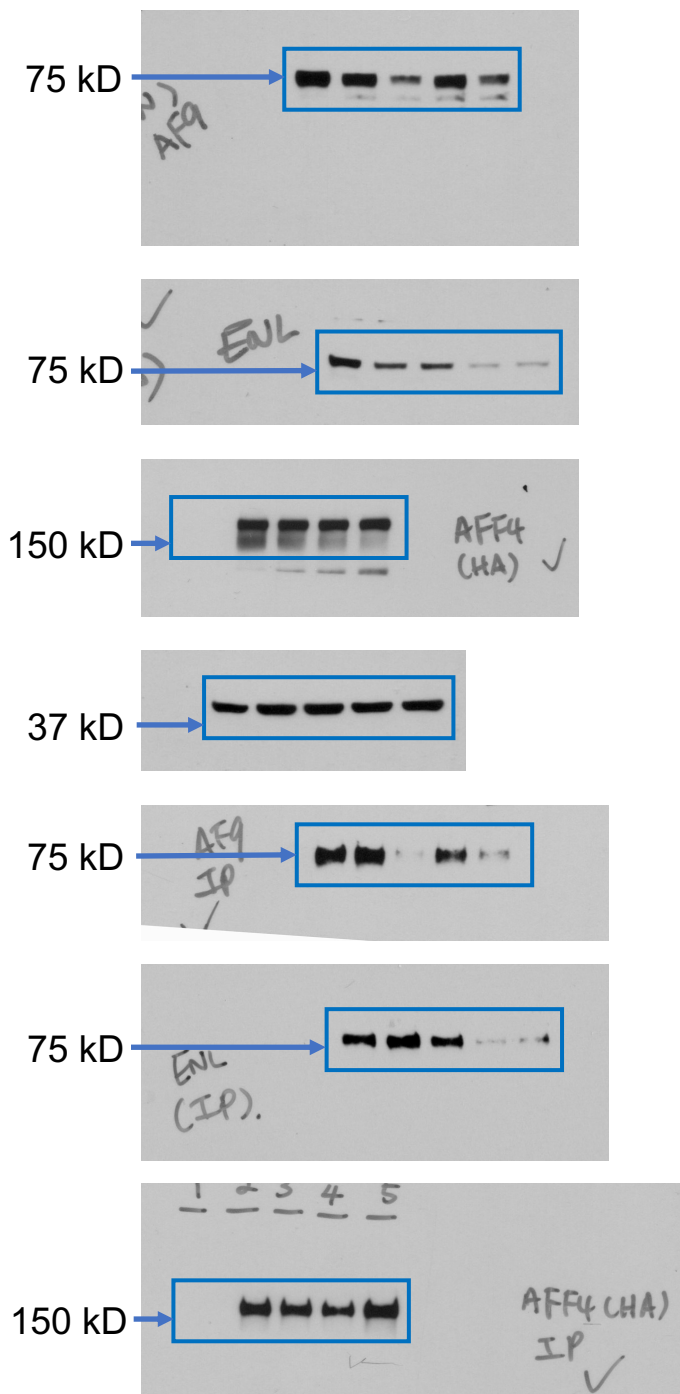

**Fig.2c**

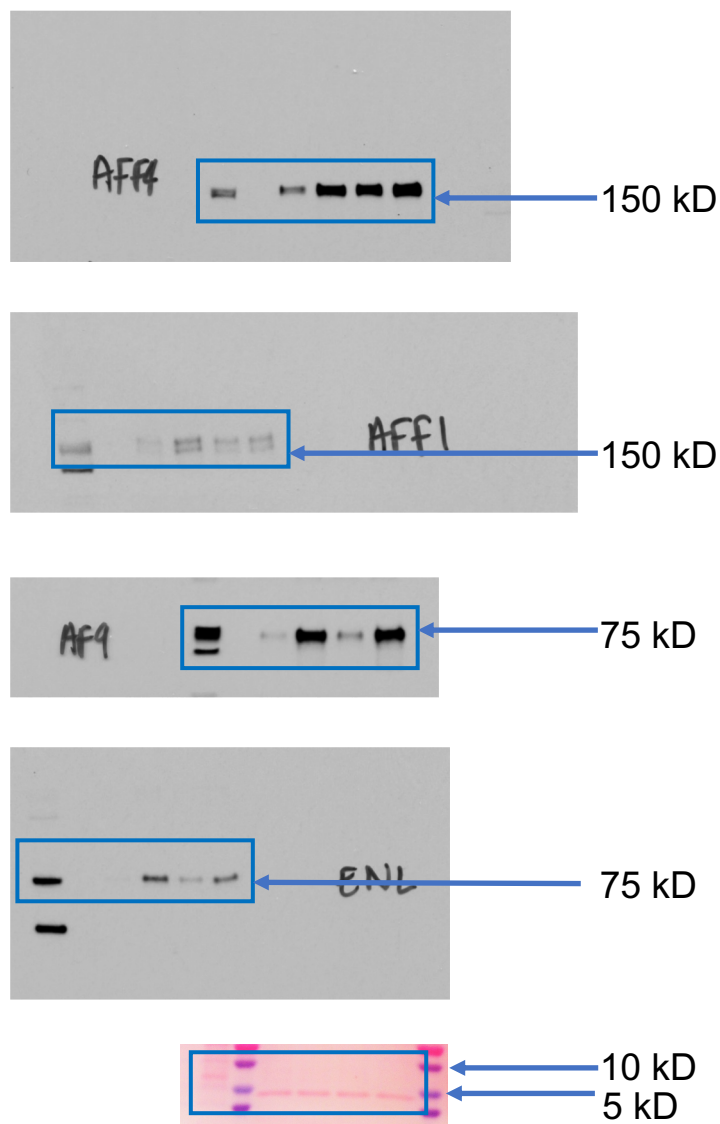

**Fig.2d**

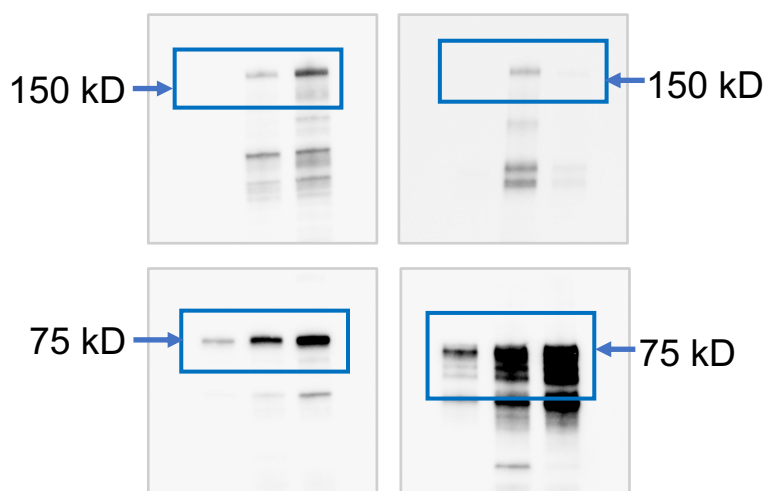

**Fig.2e**

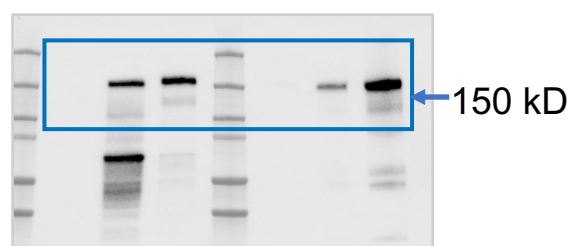

**Fig.2f**

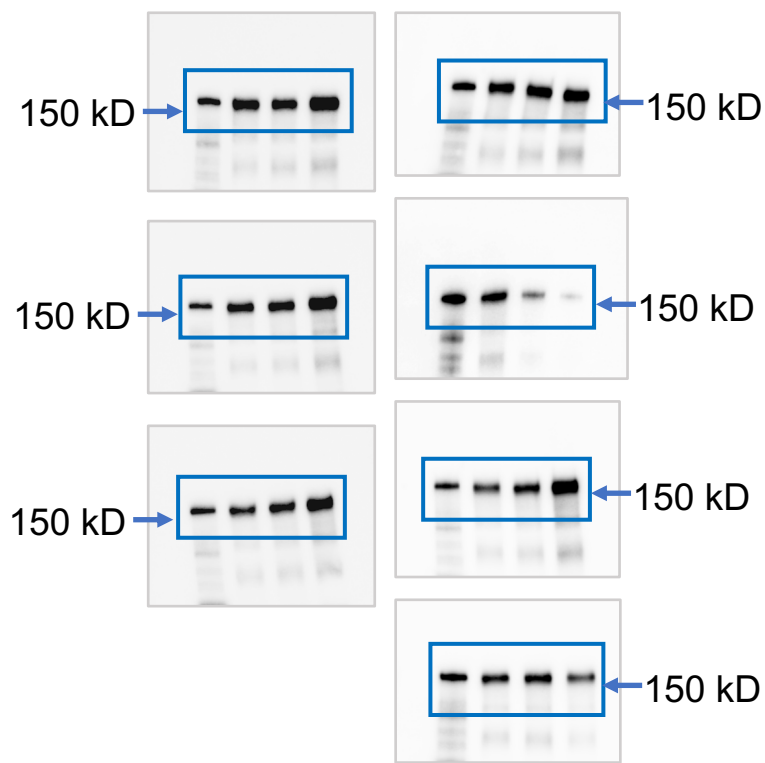

**Fig.2g**

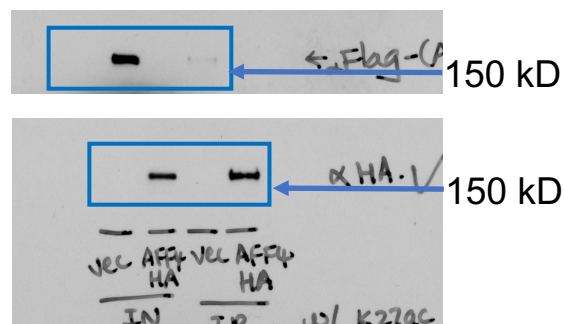

**Fig.3a**

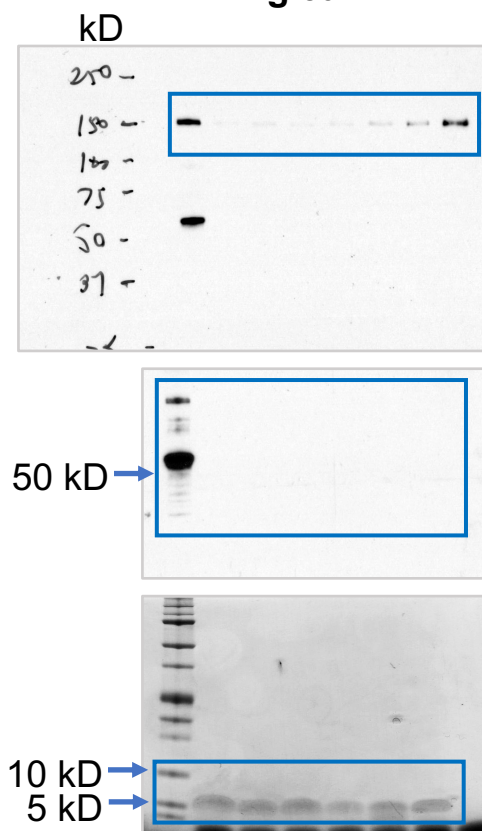

**Fig.3c**

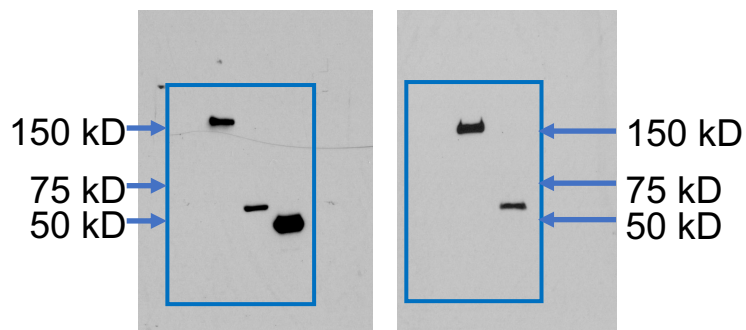

**Fig.3d**

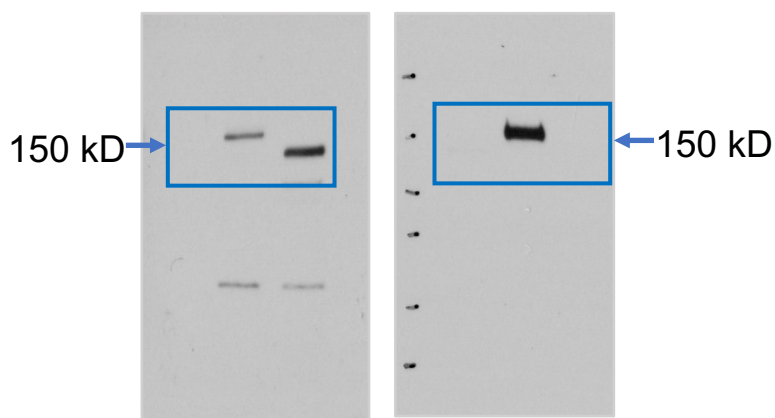

**Fig.3e**

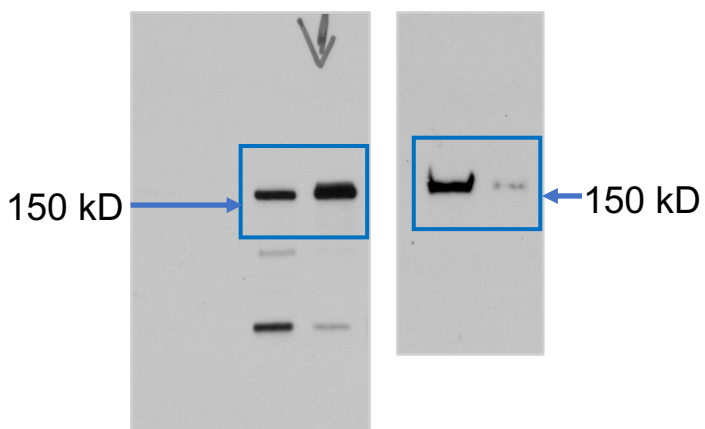

**Fig.5d**

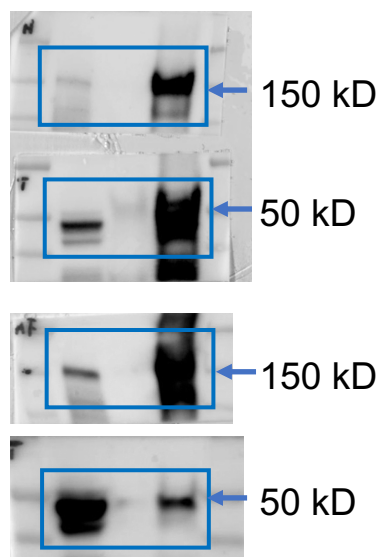

**Fig.6a**

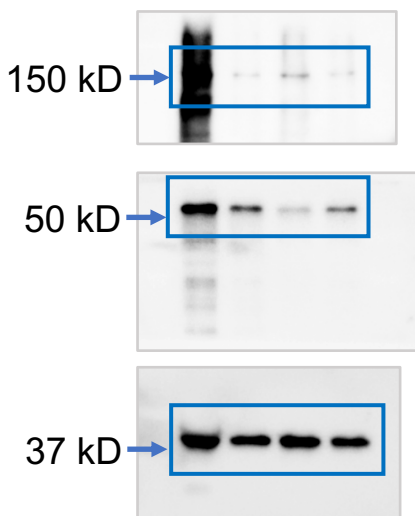

**Fig.6e**

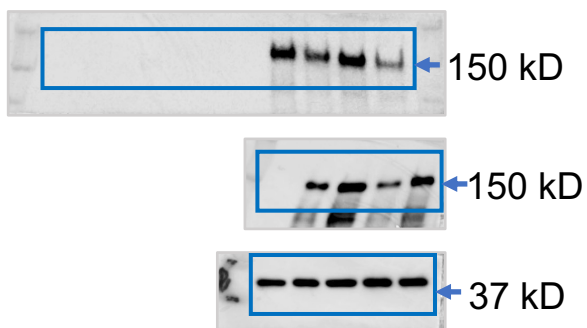

**Fig.6f**

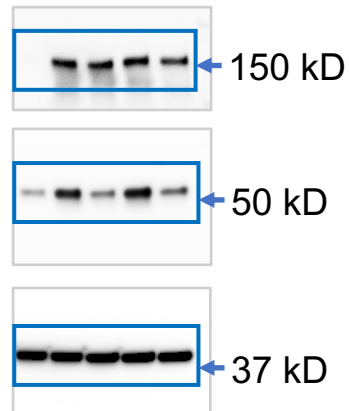

**Fig. S2b**

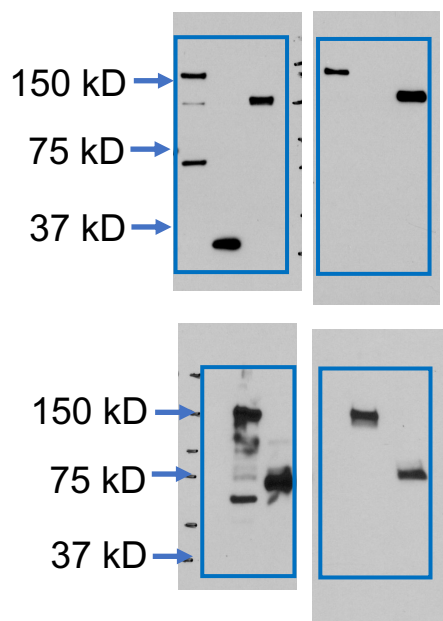

**Fig. S2c**

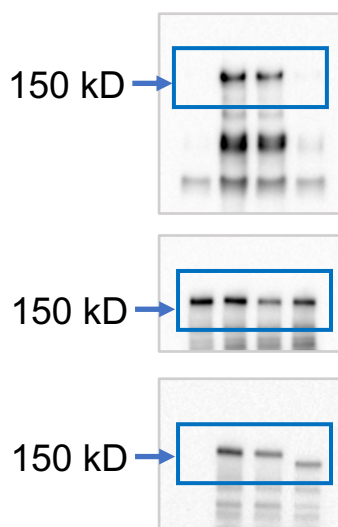

**Fig. S2d**

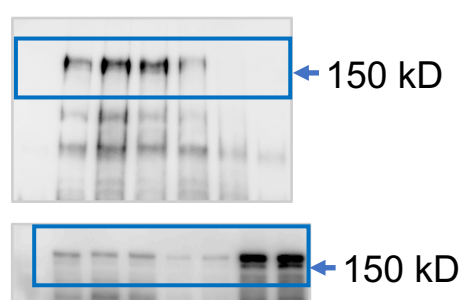

**Fig. S5a**

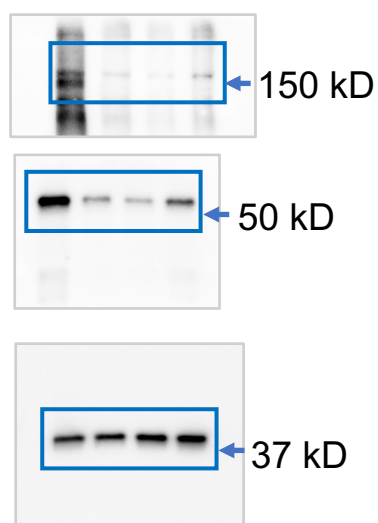

Supplement: Supplementary file 3 — Supplementary Data 1 [file 42003_2020_898_MOESM3_ESM.pdf]
