# Supplementary material for: Acetylation of histone H3K27 signals the transcriptional elongation for estrogen receptor alpha
Source: Commun Biol. 2020 Apr 7;3:165. doi: 10.1038/s42003-020-0898-0 (PMC7138820; doi:10.1038/s42003-020-0898-0)
Supplement: Supplementary file 7 — Reporting Summary [file 42003_2020_898_MOESM7_ESM.pdf]

nature research

Corresponding author(s): Qin Feng

Last updated by author(s): 03/14/2020

Reporting Summary

Nature Research wishes to improve the reproducibility of the work that we publish. This form provides structure for consistency and transparency in reporting. For further information on Nature Research policies, see Authors & Referees and the Editorial Policy Checklist.

Statistics

For all statistical analyses, confirm that the following items are present in the figure legend, table legend, main text, or Methods section.

n/a	Confirmed	
☐	☒	The exact sample size (*n*) for each experimental group/condition, given as a discrete number and unit of measurement
☐	☒	A statement on whether measurements were taken from distinct samples or whether the same sample was measured repeatedly
☐	☒	The statistical test(s) used AND whether they are one- or two-sided<br>*Only common tests should be described solely by name; describe more complex techniques in the Methods section.*
☒	☐	A description of all covariates tested
☒	☐	A description of any assumptions or corrections, such as tests of normality and adjustment for multiple comparisons
☐	☒	A full description of the statistical parameters including central tendency (e.g. means) or other basic estimates (e.g. regression coefficient) AND variation (e.g. standard deviation) or associated estimates of uncertainty (e.g. confidence intervals)
☒	☐	For null hypothesis testing, the test statistic (e.g. *F*, *t*, *r*) with confidence intervals, effect sizes, degrees of freedom and *P* value noted<br>*Give P values as exact values whenever suitable.*
☒	☐	For Bayesian analysis, information on the choice of priors and Markov chain Monte Carlo settings
☒	☐	For hierarchical and complex designs, identification of the appropriate level for tests and full reporting of outcomes
☐	☒	Estimates of effect sizes (e.g. Cohen's *d*, Pearson's *r*), indicating how they were calculated

Our web collection on statistics for biologists contains articles on many of the points above.

Software and code

Policy information about availability of computer code

Data collection	UCSC genome cancer browser, cBioPortal for Cancer Genomics
Data analysis	MACS, Cistrome analysis pipeline, GREAT (Genomic Region Enrichment Annotation Tool), DAVID bioinformatics resources, ProbeFinder, Multalin, XDS software package, Phenix.autosol, Coot, Phenix.refine

For manuscripts utilizing custom algorithms or software that are central to the research but not yet described in published literature, software must be made available to editors/reviewers. We strongly encourage code deposition in a community repository (e.g. GitHub). See the Nature Research guidelines for submitting code & software for further information.

Data

Policy information about availability of data

All manuscripts must include a data availability statement. This statement should provide the following information, where applicable:

- Accession codes, unique identifiers, or web links for publicly available datasets
- A list of figures that have associated raw data
- A description of any restrictions on data availability

The ChIP-seq data presented in Figure 4 and 5 are available at NCBI's gene expression omnibus (GEO, accession number XXXX, will be updated before publication). The protein structure data presented in Figure 3f is available in PBD protein data bank with Accession number XXX (will be updated before publication). All other relevant data generated or analyzed in this study are included in this article and the supplementary information.

Field-specific reporting

Please select the one below that is the best fit for your research. If you are not sure, read the appropriate sections before making your selection.

☒ Life sciences ☐ Behavioural & social sciences ☐ Ecological, evolutionary & environmental sciences

For a reference copy of the document with all sections, see nature.com/documents/nr-reporting-summary-flat.pdf

Life sciences study design

All studies must disclose on these points even when the disclosure is negative.

Sample size	Sufficient sample sizes were chosen for each experiment to determine whether the outcome was statistically significant.
Data exclusions	No data were excluded.
Replication	Cell growth samples were at least triplicated. ChIP-qPCR experiments were duplicated and repeated for at least two times. RT-qPCR experiments were duplicated and repeated for three times.
Randomization	Randomization was not applicable in this study.
Blinding	Blinding was not applicable in this study.

Reporting for specific materials, systems and methods

We require information from authors about some types of materials, experimental systems and methods used in many studies. Here, indicate whether each material, system or method listed is relevant to your study. If you are not sure if a list item applies to your research, read the appropriate section before selecting a response.

Materials & experimental systems

n/a	Involved in the study	
☐	☒	Antibodies
☐	☒	Eukaryotic cell lines
☒	☐	Palaeontology
☒	☐	Animals and other organisms
☒	☐	Human research participants
☒	☐	Clinical data

Methods

n/a	Involved in the study	
☐	☒	ChIP-seq
☒	☐	Flow cytometry
☒	☐	MRI-based neuroimaging

Antibodies

Antibodies used	For Western blot: anti-TFAP2C (Santa Cruz, sc-53162, 1:1000), anti-HA antibody (Sigma/Roche, 11867423001, 1:1000), anti-AFF4 (Bethyl Labs, A302-538A, 1:1000), anti-β-actin (Sigma, A2228, 1:2500), anti-ERα (Abcam, ab108398, 1:1000), anti-AFF1 (Bethyl Labs, A302-345A, 1:1000), anti-AF9 (Bethyl Labs, A300-597A, 1:1000), anti-ENL (Bethyl Labs, A302-267A, 1:1000), anti-Flag (Sigma, A8592, 1:1000), and anti-GFP (Santa Cruz Biotechnology, sc-9996, 1:1000).<br>For ChIP-qPCR: rabbit IgG (Santa Cruz Biotechnology, sc-2027), mouse IgG (Santa Cruz Biotechnology, sc-2025), anti-H3K27ac (Abcam, ab4729), anti-AFF4 (Bethyl Labs, A302-538A), anti-RNA polymerase II (Abcam, ab817), and anti-Cylin T1 (Santa Cruz Biotechnology, sc-8127).
Validation	All antibodies were validated by the manufacturers with multiple publications referred.

Eukaryotic cell lines

Policy information about cell lines

Cell line source(s)	All cell lines were initially purchased from ATCC.
Authentication	All cell lines were authenticated using morphology and STR profiling.
Mycoplasma contamination	All cell lines were tested negative for mycoplasma contamination by PCR-based method.
Commonly misidentified lines (See ICLAC register)	None

ChIP-seq

Data deposition

☒ Confirm that both raw and final processed data have been deposited in a public database such as GEO.

☒ Confirm that you have deposited or provided access to graph files (e.g. BED files) for the called peaks.

Data access links<br>*May remain private before publication.*	As this is the initial submission, the data deposition is pending and the data access links will be provided during revision.
Files in database submission	The ChIP-seq raw data will be deposited as fastq files, and the processed files will be deposited as bigwig files.
Genome browser session<br>(e.g. UCSC)	This will be provided when submitting a revised manuscript.

Methodology

Replicates	Because we performed the ChIP-seq in Active Motif Inc, the samples have no replicates due to the high cost. But we performed pilot ChIP-seq to validate each antibody prior to the formal ChIP-seq experiment. We also sequenced Input sample as a background control. Additionally, the important peaks related to this study were all validated by ChIP-qPCR.
Sequencing depth	The 75-nt sequence reads generated by Illumina sequencing (using NextSeq 500) are mapped to the genome using the BWA algorithm with default settings. ChIP-seq samples yielded 31-40million single-ended reads. Only reads that pass Illumina's purity filter, align with no more than 2 mismatches, and map uniquely to the genome are used in the subsequent analysis.
Antibodies	anti-histone H3K27ac (Active Motif, 39133), anti-AFF4 (Bethyl Labs, A302-538A).
Peak calling parameters	Peak calling was performed using MACS with the following settings: mfold 10-30, p value 10e-13
Data quality	Compared to the default MASC setting for peak calling (which is mfold 10-30, p value 10e-5, we used a much more stringent criterion and therefore resulted in very robust peaks, with 90% of the peaks with higher than 5 fold enrichment over background.
Software	MACS, Cistrome analysis pipeline, GREAT (Genomic Region Enrichment Annotation Tool), DAVID bioinformatics resources.
